# Challenges in dementia care: comparing key issues from Brazil and the United Kingdom

**DOI:** 10.1590/1980-57642020dn14-030003

**Published:** 2020

**Authors:** Helen Durgante, Milena Lucía Contreras, Tamara Backhouse, Angelique Mavrodaris, Michele Gomes Ferreira, Debora Lee Vianna Paulo, Marcus Vinicius Alves, Larissa da Silva Serelli, Leonardo Cruz de Souza, Naoko Kishita

**Affiliations:** 1 Departamento de Psiquiatria, Universidade Federal de São Paulo - São Paulo, SP, Brazil.; 2 School of Health Sciences, University of East Anglia - Norwich, United Kingdom.; 3 Cambridge Institute of Public Health, University of Cambridge - Cambridge, United Kingdom.; 4 Faculdade de Medicina, Universidade Federal de Minas Gerais - Belo Horizonte, MG, Brazil.; 5 Faculdade de Ciências Médicas, Universidade Estadual de Campinas - Campinas, SP, Brazil.; 6 Departamento de Psicobiologia, Universidade Federal de São Paulo - São Paulo, SP, Brazil.

**Keywords:** dementia, health care, health programs and plans, cross-cultural comparison, healthcare systems, demência, assistência à saúde, planos e programas de saúde, comparação transcultural, atenção à saúde

## Abstract

The United Kingdom-Brazil Dementia Workshop took place in July 2019 in the city of Belo Horizonte, MG, Brazil, with an interdisciplinary group of health and care professionals from the United Kingdom and from Brazil to address challenges in diagnosis, public perception and care of dementia. The aim of this article is to present the results identified in relation to challenges in the care of dementia, including recommendations that could potentially guide local and State/Municipal authorities and care services for people with dementia in the future. Four key issues were prioritised to identify challenges and generate possible solutions in Brazil and the United Kingdom: I) limitations of current health systems; II) continuous and long-term support for family carers (pre-diagnosis, mourning); III) support for people with advanced dementia and end-of-life care; IV) support for people with young-onset dementia. In both countries, carers feel left without post-diagnostic support; information on the progression of dementia is lacking and some people do not even have a specific diagnosis; encouraging and providing training for carers best manage some of the symptoms is imperative; preparation for end of life care and support carers after the death of their loved ones remains highly needed; strengthening services and qualification of health professionals, also creating protocols to guide dementia-related services represent a common challenge to overcome. The authors outline recommendations according to the issues identified to assist future formulation of adequate policies and services for people with dementia and carers.

## RESUMO.

O *United Kingdom-Brazil Dementia Workshop* aconteceu em julho de 2019 na cidade de Belo Horizonte, Minas Gerais, Brasil, com a presença de grupo interdisciplinar de profissionais de saúde e assistência do Reino Unido e Brasil, para abordar desafios no diagnóstico, percepção pública e cuidado à pessoa com demência. O objetivo do presente artigo foi apresentar os resultados encontrados em relação aos desafios no tratamento da demência, incluindo recomendações que possam potencialmente orientar autoridades locais e estaduais/municipais e serviços de atenção à pessoa com demência. Quatro questões-chave foram priorizadas para identificar desafios e gerar possíveis soluções no Brasil e no Reino Unido: I) limitações atuais dos sistemas de saúde; II) suporte contínuo e de longo prazo para cuidadores familiares (pré-diagnóstico, luto); III) apoio a pessoas com demência avançada e cuidados no fim de vida; IV) apoio a pessoas com demência precoce. Em ambos os países, cuidadores se percebem sem apoio pós-diagnóstico; faltam informações sobre a progressão da demência e algumas pessoas sequer têm diagnóstico específico; encorajar e providenciar treino para cuidadores melhor lidarem com sintomas é imperativo; preparação para cuidados no fim da vida e apoio aos cuidadores após morte de familiares permanecem necessários; fortalecer os serviços e qualificação de profissionais de saúde, além de elaborar protocolos para guiar serviços relacionados à demência, é desafio comum a ser superado. Os autores apresentam recomendações quanto às questões identificadas para auxiliar na futura formulação de políticas públicas e serviços para pessoas com demência e cuidadores.

## INTRODUCTION

The United Kingdom-Brazil Dementia Workshop, co-hosted by the Universidade Federal de Minas Gerais (UFMG) and the University of East Anglia (UEA), took place in Belo Horizonte/MG/Brazil during two days of July 2019. Entitled “Challenges in Cognitive Impairment and Dementia: (Mis)perceptions, (Mis)diagnosis and Care Management”, the workshop brought together junior researchers, health/care practitioners and senior clinicians from Brazil and the UK to share knowledge about the key challenges in dementia care. The workshop provided an opportunity to discuss similarities and differences between both countries and aimed to facilitate the collaborative design of potential recommendations to address challenges in diagnosis, public perception and care of dementia.

Key demographic and geographical differences between the UK and Brazil exist and provide context. Brazil has a larger population (over 209 million people in 2018; UK approximately 66 million in 2018), and has a larger territory (8.5 million km^2^), compared to the UK (243,000 km^2^). In contrast, the UK Gross Domestic Product (GDP) per capita in 2017 was much higher (US$40,158) than that of Brazil (US$14,283) for the same period.^1^

In both countries, however, populations are ageing and the prevalence of diseases connected with ageing is increasing. Dementia presents a major public health challenge across both nations. Currently, it is estimated that 35.6 million people are living with dementia globally and numbers are expected to almost double every 20 years, with 71% of cases being in low or middle-income countries by 2050.^2^ Dementia was the leading cause of death (12.7% of all deaths recorded 533,253) in England and Wales in 2017,^3^ while in Brazil Alzheimer’s Disease (AD), in particular, was the fourth cause of death.^4^

Changes in cognition and dementia progression are often associated with social and physical effects, mortality and impacts on quality of life^5^ and are associated with increased hospitalisation rates and functional decline.^6^ As a result, the costs of caring for people with dementia (PwD) are high, without taking into account the intangible costs - the distress and loss that PwD, their families and carers experience.^7^ ^,^ ^8^

This article aims to describe and share knowledge of differing and specific challenges in dementia care faced by both countries to produce tangible outputs and recommendations that could further inform the development of local services. Four key issues of dementia care were prioritised: limitations of current healthcare systems; long-term support for family carers; support for people with early-onset dementia and support for people with advanced dementia and end-of-life care. A summary of the main challenges and recommendations made by workshop participants are provided in Table 1.

**Table 1.** Comparison of dementia care challenges and recommendations (Brazil and UK).

Common issues	Recommendations
Healthcare systems	
Decentralised accountability of the Government for providing care to reduce costs	Advocate that governments increase public funding allocated to dementia services
Quality regulation of services outcome-driven, perceived benefits are questionable	Implement systems to protect and ensure safety/quality of services leading to tangible actions
Decentralised accountability of the Government for providing care	Encourage governmental engagement and investment in care provision
Limited availability of home-care service	Increase capacity-building and personnel for home-care visits and health centres
Continuous support/long-term support for family carers	
Availability of health/social care support services vary according to regions	Develop community-based services specific to local needs and aligned with the third sector
Lacking post-diagnosis support	Encourage integrated support groups from communities, universities and public health services
Providing formal training to disseminate information for family carers and health professionals	Support collaborative work through public services and third sector to reach families and increase dementia awareness
Support for people with YOD	
Service design not appropriate for specific needs of YOD	Develop multiprofessional collaborative networks to expand consultation and tailor appropriate services
Pathways into care: Many different specialists and long time to receive a final diagnosis within the public system	Improve recognition/knowledge of YOD for primary care physicians and non-specialists where such facilities may be scarce
Difficulty in diagnosis/mis/under-diagnosis and high overlap with other mental health conditions	Use well-established decision-making tools designed to guide diagnosis and raise awareness of key red flags to diagnosis
Support for people with advanced dementia/end-of-life care	
Funding for and affordability of quality care services	Advocate for investments in public policies and long-term care
Responsibility for who should care of PwD unclear	Support active voice of service users and family members to start public discussion
Availability of quality care services for PwD and families	Advocate for investments in public policies and funding allocation for care services for PwD
Not sufficient dementia specific bereavement support/only generic palliative care	Advocate for investment in public policies and funding allocation to structure bereavement support/palliative care groups

YOD: Young Onset Dementia; PwD: people with dementia.

### Limitations of current healthcare systems

The Brazilian Unified Public Health System (SUS) is comparable to the initial structure of the National Health System (NHS) in the UK, which had a tripartite system, including Primary care, Hospital and Community services for the provision of health care through general taxation.^9^ ^,^ ^10^ In England, currently two service infrastructures are in place - Health and Social Care - which coordinate and provide services. The Care Quality Commission regulates services,^11^ that is, registers, monitors, inspects and rates services, and takes action to protect service users. In Brazil, the National Sanitary Surveillance Agency (ANVISA) functions as a health regulatory agency; conducting risk analyses and inspections of health-related services and sanitary control.^12^ Private insurance companies/services are monitored by the National Agency of Supplementary Health.^13^ Although both countries have a system in place to ensure the quality of care, the integrity of services provided by regulatory agencies is questionable on the basis that services may be outcome-driven as opposed to focusing on service users’ well-being.

Nowadays, the structure of both health systems has shifted towards more decentralised accountability for providing care to facilitate services integration and efficiency. In the UK, funding from the Department of Health is transferred to Clinical Commission Groups, which identify local health demands, plan and contract health services from various public and private organisations, or charities, following a market-based rationale.^9^ Currently, over 83% of the total expenditure has been allocated to public health in the UK^14^ and, despite this, home care (under the Social Care System) covers a maximum of four visits a day for advanced dementia, and no overnight service is available.

In the Brazilian scenario, the number of home-care visits is limited to one-two on a weekly basis, depending on staff availability, geographic regions and competing epidemiological demands (Zika virus, Malaria, etc.), making dementia a sub-priority. Low levels of formal education and inadequate dementia training for community health agents is a critical issue. Private insurance plans provide 24-hour home-care and specialised consultations.

Nonetheless, even in the Brazilian private system there is a lack of specialised health professionals and adequate structures. Most of specialised centres for dementia care in Brazil are associated with public universities in larger cities. These centres have well trained staff caring for PwD but only provide support for a fraction of PwD. This illustrates a fragmentation of health services in Brazil with less than 50% of public budget directed to health where over 70% of the population use public health services.^15^

There is growing recognition of the need to establish the integrated care system for PwD worldwide. This requires an effort in training primary care professionals beyond diagnosis and pharmaceutical management. However, lack of opportunities for training and mentorship and challenges of adjusting to a new way of learning (i.e., a biomedical rather than person-centred focus) among general practitioners often become barriers to the implementation of integrated care at an individual level in addition to organisational complexity seen in both countries.^16^

### Continuous support/long-term support for family carers (pre-diagnosis, grief)

Caring for PwD is physically and mentally exhausting. Family carers often feel burdened, isolated or stressed; with some experiencing clinically significant depression.^17^ ^,^ ^18^ Burnout and the likelihood of morbidity increases, particularly as carers get older.^19^

During the workshop, it was identified that family carers in both countries face similar challenges, feeling left without sufficient post-diagnostic support. Particular concerns were raised for patients without a diagnosis, as families struggle to plan and prepare for the future, while the lack of information on dementia progression was also highlighted. Also, the provision of health and social care services for PwD and families varies across regions, resulting in unequal access and services not tailored to specific needs.

In the UK, there are approximately 700,000 informal carers of PwD.^20^ ^,^ ^21^ While services such as admiral nurses exist to provide support for carers, not all families can access these and the lack of skilled professionals able to address the diverse needs of families remains an issue.^22^ The voluntary sector and charities in the UK contribute to raising awareness and supporting families living with dementia. Dementia/Memory Cafes, helplines, peer support groups and training sessions are widely used to provide information, reduce isolation and increase emotional support among families and carers.^23^

In Brazil, research on estimates of informal carers is scarce and data only regards specific samples,^24^ ^,^ ^25^ despite the prevalence of dementia being 1.6% for the 65-69 age group, increasing to 38.9% in the age group over 84 years.^26^ Due to the absence of an adequate formal support network, family members generally assume primary caregiving responsibilities extending over long periods.^27^ Many family carers have little knowledge about dementia and how to deal with issues experienced by PwD on a daily basis. In Brazil, dementia is mostly considered part of normal ageing; this misconception and the lack of healthcare services specialised in dementia back the belief that families should be responsible for PwD.^28^

Creating Dementia/Memory Cafes in Brazil could be one way of bringing family carers closer to other carers and dementia care professionals. Dementia/Memory Cafes have been effective in the UK^23^ and could potentially be adopted in Brazil where the sense of community is strong. In Brazil, cultural values/beliefs reinforce the perception that children should look after their parents in advanced ages as part of familial duties.^28^ In both countries, the provision of training for family carers and health professionals was identified as a key enabler of improved dementia care.

### Support for people with young-onset dementia

‘Young-onset dementia’ (YOD), described as dementia diagnosed under 65 years, is poorly recognised.^29^ YOD can present with more severe symptoms and rapid progression compared to other dementias. Lacking global prevalence figures, atypical presentation and the majority of dementia services being focused on the needs of older people results in delays in diagnosis and limited provision of age-appropriate services. People with YOD are often parents, managing work and financial pressures and caring for older family members,^30^ thus presenting different needs and service requirements in comparison to people with late-onset dementia (LOD).

The features of YOD have significant impacts on spouses^31^ and children^32^ who are likely to be the primary resource for care. Carers of people with YOD often experience difficulties in coping with behavioural changes specific to YOD^33^ and levels of care burden are reported to be much higher than that of carers of people with LOD.^33^ These challenges are apparent both in the UK and in Brazil.

Atypical presentations, including new-onset depression, behavioural or cognitive changes are common in YOD. Difficulty in diagnosis/under-diagnosis, overlap with psychiatric syndromes and the stigma or stereotype of assumptions that a young person has behavioural or social issues rather than YOD are common issues in both countries. This complexity often results in diagnostic delays or misdiagnosis, which causes additional stress and frustration for families.^30^ ^,^ ^34^

In Brazil, there is no specific service tailored to YOD. Users of public health services have less access to medical care, pharmacological coverage and access services at more advanced stages of dementia compared to users of the private sector.^35^ In the UK, evidence indicates that young people with signs of dementia see a minimum of two - and some up to five - different specialists before receiving a final diagnosis while pathways into care are disorganised and unsystematic. The majority of younger people in the UK continue to be assessed and diagnosed in mental health-led memory clinics where limited access to other disciplines is well documented.^36^

Improving awareness, recognition and knowledge of YOD within health and care services and communities and developing responsive age-appropriate pathways of care are fundamental ambitions in both countries. Initiatives such as a new decision-making tool developed by the Young Dementia Network UK, which is designed to guide diagnosis and raise awareness,^37^ and remote-area initiatives to expand consultation services using videoconferencing and telementoring^38^ are promising examples of progress.

### Support for people with advanced dementia and end-of-life care

Significant functional and cognitive decline characterises the advanced stage dementia.^39^ As people progress towards advanced dementia, they need more assistance with activities of daily living and can exhibit dementia-related behaviours, creating a higher level of burden on the carer, and a greater risk of institutionalisation.^39^ ^,^ ^40^

In the UK, family carers, home-care workers or transitions to care homes attempt to address these care needs. The term ‘care home’ in the UK refers to communal living settings offering accommodation and 24-hour assistance with personal care and other daily tasks, some homes have qualified nursing staff on duty at all times (nursing homes) and others have no nursing staff (residential homes). Home-care workers provide support at the person’s own home. Care home entry is common for PwD in the UK.^41^ EOL care can occur in hospices (specialist palliative care settings), care homes or family settings.

There is a general expectation that social care will be paid for by the government. Social care services are means tested and the government pays for people who have limited resources; if there are demonstrable health care needs, which meets a predetermined threshold, the NHS will cover the costs of care.^42^ Dementia is a condition that requires both health and social care support, however, the health component is often overlooked and social care funding from the government is limited.^43^ Although family carers are common, there is no expectation that family members should be the only carers in advanced dementia or at EOL.

In Brazil, the subpopulation of people with advanced dementia is not adequately characterised and estimated, their clinical and sociodemographic context are unknown, as well as the sociodemographic profile of carers.^39^ People with advanced dementia show a high morbidity profile, have low income, and limited assistance provided by formal carers, while the main carers are family members, especially unemployed daughters.^39^

In Brazil, there is no expectation that the government should provide care and families are expected to care for their loved ones.^29^ Institutions that set-up partnerships with public funding, the government contribution is limited to core medical services or drug supply.^44^ In addition, the presence of dementia-related behaviours are often misinterpreted in public, creating shame, stigma, stress, and resulting in those with disruptive behaviours not being seen by health professionals, but instead being hidden away by the family carers.^45^ Short, infrequent visits by healthcare professionals are available for PwD, however, competing demands on health agents leads to limited appointments.

In the UK, the proportion of the population older than 65 years living in care homes is 5.1% and in Brazil the estimated prevalence is from 0.46 to 1.0%.^46^ This relatively low use in Brazil could be due to the limited number of care homes and the stigma attached to living in these institutions.^44^ Private care homes are more common and available for those who can afford their extremely high costs.

Although some support may be provided by the NHS, in the UK, most bereavement support is provided by charities such as the Alzheimer’s Society and Cruse Bereavement Care in the form of information factsheets and free bereavement counselling.^47^ In Brazil, there is no formal bereavement support available. In some health services or associations, there are managed bereavement support groups, particularly in palliative care groups, but they are not services offered as a responsibility of the health system.

The United Kingdom-Brazil Dementia Workshop provided room for in-depth discussions and potential recommendations pertinent to health and care professionals, researchers, and decision-makers across countries. Findings from both countries highlighted distinct challenges but also converged to inform discussion and generation of overarching approaches to address these challenges.

Providing a timely and accurate diagnosis where possible is fundamental to empowering PwD and families to access treatment, support and plan for the future.^48^ Raising awareness and advocating for the specific needs of PwD through service users/peer-to-peer (as a highly motivated group) interacting with the third sector and health and care professionals could be effective in raising awareness, disseminating knowledge and refuting stigma.

Multidisciplinary assessment to establish YOD diagnosis and facilitate integration between specialists and join-up with a broad range of services (including third sector) could enable PwD and their families to access appropriate support at home and in communities. People affected by YOD and family organisations should be supported to participate in policy making and service planning. Researchers from Higher Education Institutions could also contribute to disseminate knowledge, solve doubts and myths/stigma about dementia.

Capacity building of health professionals working across hospitals and Primary Care is an important investment to make as these are usually the gateways for services to the majority of users of Public Health Systems. Integrating and building capacity could be an effective and responsive public health approach to ensure PwD, carers and families can access timely support and services relative to their specific needs. Strengthening ties with community leaders and non-profit and/or religious institutions may be a way forward as they have greater access to people from poorer communities who generally lack information about dementia. Creating and increasing funding opportunities and international collaborations could build accessible knowledge exchange, provide resources to non-profit organisations, train community leaders and support family carers. One way to improve care for PwD could be through community activation through churches and cross-sectoral action - public-third sector initiatives.

This article aimed to provide potential recommendations to address challenges in diagnosis, public perception and care of dementia in Brazil and the UK. Therefore, the implementation of these recommendations is beyond the scope of this paper. However, we hope these findings will aid further collaborative thinking on designing initiatives and implementing approaches across health and care settings, local authorities, charities and other sectors; embedding and strengthening the culture of co-design of assessment approaches, services and care planning with PwD, families and carers, hence improving cross-sectoral action to include family carers’ needs, particularly those with fewer financial resources, in the formulation of services for PwD.
